# Co-designing an Integrated Health and Social Care Hub With and for Families Experiencing Adversity

**DOI:** 10.5334/ijic.6975

**Published:** 2023-04-05

**Authors:** Teresa Hall, Sarah Loveday, Sandie Pullen, Hayley Loftus, Leanne Constable, Kate Paton, Harriet Hiscock

**Affiliations:** 1 Centre for Research Excellence in Childhood Adversity and Mental Health, Centre for Community Child Health, Murdoch Children’s Research Institute, AU; 2 Innovation, Design & Communications, Manager, IPC Health, AU

**Keywords:** co-design, family adversity, integration, hub models

## Abstract

**Introduction:**

Integrated care research often fails to adequately describe co-design methods. This article outlines the process, principles and tools to co-design an integrated health and social care Hub for families experiencing adversity.

**Research methods:**

The Child and Family Hub was co-designed in four stages: (1) partnership building and stakeholder engagement, (2) formative research, (3) persona development and (4) co-design workshops and consultations. Local families, community members and intersectoral practitioners were engaged at each stage. The co-design workshops employed a human-centred design process and were evaluated using the Public and Patient Engagement Evaluation Tool (PEET).

**Results:**

121 family participants and 80 practitioners were engaged in the Hub’s co-design. The PEET highlighted the co-design team’s satisfaction achieved by community members working alongside practitioners to generate mutual learning. Resourcing was a key challenge.

**Discussion:**

Human-centred design offered a systematic process and tools for integrating formative evidence with lived and professional experience in the Hub’s co-design. Applying community engagement principles meant that a diverse range of stakeholders were engaged across all stages of the project which built trust in and local ownership of the Hub model.

**Conclusion:**

Co-design research with families experiencing adversity should attend to language, engagement methods, team composition and resourcing decisions.

## Introduction

### Background

Participatory approaches to health service research and development are now ubiquitous [1,2,3]. Participation is best viewed as a continuum of approaches to promote the active and meaningful involvement of people in decisions affecting their health and health care [4,5,6,7,8,9]. Co-design is one method for enabling people with lived and professional experience of health services to participate in the design, delivery and evaluation of these services [4].

Co-design methods are increasingly used to solve complex problems in health care and health system management in Australia and globally [7,10,11]. Co-design is advanced as a way of enhancing the substantive (i.e., quality of research), instrumental (i.e., translation to practice), normative (i.e., intrinsic value) and political (i.e., social change) agendas of research and practice [4,8,12,13,14]. An emerging body of evidence has shown that the participation of service users in the development and delivery of health services can improve users’ health outcomes, health behaviours, experiences of care and foster a sense of empowerment [6,15,16,17]. In Australia – the setting for this study – recent policy directives including the Mental Health Productivity Commission and the Victorian government’s commission into mental health position people with lived experience at the heart of responses to mental health and child health inequity [18,19,20], mobilising these normative and political rationales to co-design.

### Problem statement

Co-design is a term used to describe a wide range of processes and practices with varying degrees of stakeholder engagement [10,16]. This is problematic for several reasons. First, this lack of standardised definition does not distinguish between extensive, involved co-design processes and cursory, tokenistic stakeholder engagement which means that “almost everyone seems to be doing [co-design]” [7]. This lack of specificity is particularly problematic in an integrated care context when service users are encountering complex health and social challenges that require integrated responses from practitioners across sectors and professional backgrounds. Active and meaningful engagement of these varied stakeholders requires a systematic strategy, resourcing and time that is often not undertaken with more cursory forms of ‘co-design’.

Second, few research reports have sufficient detail on the co-design methods adopted for large health innovations to enable them to be replicated. A critical missing detail is how research evidence, practice knowledge and lived experience are merged in the co-design process. This is an important oversight because of the inherent tension between design processes that embrace iteration, ambiguity and rapid prototyping and the requirement of health service research to specify the project design in detail when applying for project funding [10]. This ideological tension has been consistently identified as a challenge to effective co-design processes involving diverse stakeholder groups [12,16].

Third, there is a lack of research about co-designing with families experiencing adversity. Family adversity includes a range of experiences such as childhood maltreatment (e.g., physical, verbal or sexual abuse), household dysfunction (e.g., parental mental illness, family substance abuse), community dysfunction (e.g., witnessing physical violence, discrimination), peer dysfunction (e.g., stealing, bullying) and socio-economic deprivation [21]. Families experiencing adversities may require integrated care because adversities tend to cluster and cannot be managed by single sectors alone. Further, these families remain a priority group for many health research and innovation projects despite well-documented social, psychological and physical barriers to their engagement in research and design processes [22,23]. Previous co-design research with adults experiencing adversity (i.e., mental health challenges, housing insecurity) underscores the need for continuous reflection on and countering of power imbalances that manifest in the process [12,13,24]. A recent integrated care study examined co-design principles for adults returning to the community from jail [25]. However, this study did not detail the methods of co-design. Specific co-design methods that incorporate the needs and preferences of both the child and family into integrated care initiatives are crucial for achieving family-centred care [26].

Hence, there is a need for integrated care research to clearly articulate its co-design methods with families experiencing adversity. This paper fills this gap by detailing the methodological “process, principles and practical tools” [7] employed to co-design an integrated health and social care Hub in Wyndham Vale in Melbourne, Australia. We define co-design as the “active involvement of a diverse range of participants in exploring, developing, and testing responses to shared challenges” [7]. We define the Hub as a centralised service that offers a range of co-located, integrated services from multiple sectors, with linkages to external services for community-based supports [27,28].

## Research methods

### Overview

**Figure 1:**
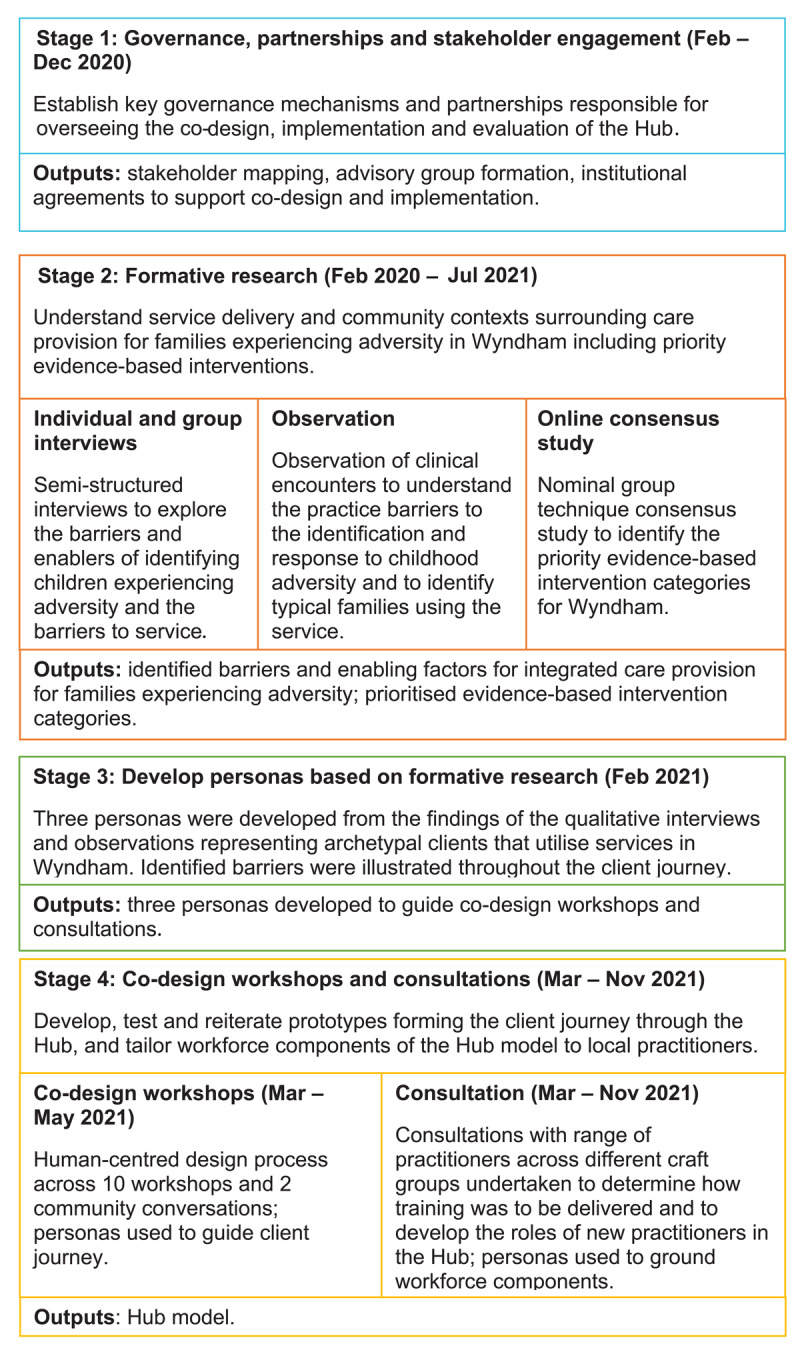
Overview of project stages supporting the co-design of the Hub.

We employed mixed methods across four project stages to undertake the co-design of the Child and Family Hub from February 2020 to November 2021 (see Figure 1). Co-design was operationalised as an iterative series of processes, core principles and design tools implemented across the planning, governance, implementation, and evaluation stages of the project [4,7]. Human-centred design was employed as an overarching framework for the project. Human-centred design is an approach to solving complex problems in which products or experiences are designed and continuously reiterated based on the perspectives, needs and preferences of the people who use them [29]. This study was approved by the Royal Children’s Hospital Human Research Ethics Committee (HREC/62129/RCHM-2020 and HREC/62866/RCHM-2020.

### Study setting

This study was conducted as part of a broader research project aiming to co-design, test and evaluate two integrated Child and Family Hub models to detect and respond to children aged 0–8 years and their families experiencing adversity in Victoria, and New South Wales in Australia [30]. This paper describes the co-design process of the Hub in Wyndham Vale, Victoria. The City of Wyndham is in the outer South-Western suburbs of Greater Melbourne and has one of Melbourne’s most culturally diverse and rapidly growing populations. More than half of Wyndham’s children aged 0–4 years have two parents who were born overseas [31]. Supporting Wyndham families is critical because approximately 25% of Wyndham’s children starting school are vulnerable in at least one development domain, compared with less than 20% children Victoria-wide [31].

### Stage 1: Governance, partnerships and stakeholder engagement

The first stage was to establish the governance mechanisms and partnerships to oversee the co-design, implementation and evaluation of the Hub. Two key local partners were engaged from the outset: IPC Health, a community health service provider, and Wyndham City Council (WCC), a local government partner. The IPC Health Wyndham Vale GP Super Clinic was selected as the Hub site because it hosts a range of primary and allied health practitioners and social services including financial counselling. While fostering these partnerships, KP mapped and approached more than 30 organisations working with families of young children in and around Wyndham Vale. Specifically, member organisations of the WCC Early Years network, WCC Child and Family Alliance and WCC Vulnerable Children’s Strategic network.

The key output of this stage was a local project advisory group with representation from health, community and education service provider organisations, local government, and a community representative with lived experience. These partnerships ensured that the project had the requisite institutional support, resources and community linkages, and was responsive to local priorities [24].

During this phase, we also established our interdisciplinary team. Our team has a mix of expertise in public health, research, health practitioner (i.e., paediatrics and nursing), lived experience of adversity, project management, design facilitation and community engagement.

#### Identification of and engagement with families experiencing adversity

Our starting point was to identify and overcome barriers to meaningful participation by families experiencing adversity [22,23]. Lived experience was prioritised as a type of expertise central to the project in all communications and research activities [7]. Valuing lived experience meant that we were reflexive about why and how much disclosure of adversity was necessary for families to engage [24]. Recruitment materials for community members specified that we were interested in engaging with caregivers experiencing adversity but would not require them to disclose their specific experiences. This approach was appropriate because we were interested in understanding and learning from families’ experiences of services rather than their personal experiences of adversity. We recruited through social media (i.e., Facebook) and known local service networks (i.e., by posting a project summary and call for participants to the WCC online project Hub), which has been shown to be effective for recruiting hard-to-reach populations [22].

#### Language and framing of the project

Creating a shared language and framing of the core project concepts (i.e., co-design and adversity) was important for these partnerships. We positioned co-design as a point on a spectrum of participatory approaches: our project involved more than a one-off consultation, but its defined scope precluded it from being fully community-led [4,7,8]. We framed the Hub co-design as comprising fixed and flexible inputs (see Table 1). The fixed inputs were those specified in the project funding agreement based on evidence reviews and program theories of integrated care for families experiencing adversity [32,33,34]. The flexible components concerned the client experience of the Hub. Early in the engagement process, we adopted lay language to describe the study. For example, ‘life challenges’ was adopted instead of ‘adversity’ and ‘conversation’ replaced ‘research or discussion’ [23].

**Table 1:** Thematic inputs, format and engagement strategy for co-design workshops and consultations.


TOPIC	INPUT TYPE	FOCUS AREA	ENGAGEMENT FORMAT	USER TESTING	STAKEHOLDERS

***Client journey***					

**Entice**	Flexible	How families first become aware of the Hub	Workshops 1 and 2	Core team testing, community conversation at shopping centre	Families experiencing adversity; Wyndham community members; health, social, family service and education practitioners

**Enter**	Flexible	How families first enter the Hub including the appeal of the physical space	Workshops 3 and 4	Core team testing, community conversation at shopping centre	Families experiencing adversity; Wyndham community members; health, social, family service and education practitioners

**Engage, Exit and Extend**	Flexible	How to create a trusted and holistic Wellbeing Coordination program at the Hub	Workshops 5, 6, and 7	Core team testing, community conversation at community centre	Families experiencing adversity; Wyndham community members; health, social, family service and education practitioners

***Workforce capabilities and infrastructure***					

**Workforce training and development**	Fixed	Preferences for delivery of practitioner training. Development of new practitioner roles within the Hub including child speech pathologist.	Workforce consultations	n/a	Health, social, family service practitioners

**Learning collaboratives**	Fixed	Preference of timing and structure of monthly learning collaboratives	Workforce consultations	n/a	Health, social, family service practitioners

**Community directory**	Fixed	Preference for presentation of community directory i.e., online or in a physical folder	Workforce consultations	n/a	Health, social, family service practitioners


### Stage 2: Formative research to understand service and community setting in Wyndham

The second stage involved extensive formative research to understand the service delivery and community contexts in which care is provided for families experiencing adversity in Wyndham [35]. We used a suite of methods to increase the participation of community members and practitioners that included individual and group interviews, observation and an online consensus study.

The practitioner interviews explored how health and social care practitioners identified children experiencing adversity and the barriers they encountered in linking families to appropriate community resources. The family interviews focused on the challenges families experienced in getting help for life challenges. The observations at IPC Health Wyndham Vale focused on the challenges families faced as expressed during a normal clinical encounter and the typical journey of these families for getting support. All interviews and observations were guided by interview/observation guides formulated to capture experiences of the challenges and opportunities in accessing and/or providing support for families in Wyndham. Interviews were audio recorded and transcribed by a transcription service. Thematic analysis was used to analyse the interview and observation data.

We also conducted an online consensus study [36] in which practitioners and families prioritised interventions for children experiencing adversity in Wyndham based on our prior evidence review [34]. This formative qualitative research provided depth to our understanding of local needs, preferences and contextual factors to ensure that the co-design built upon what was already known about and had worked before in Wyndham [37].

### Stage 3: Development of personas and client journeys based on formative research

**Figure 2:**
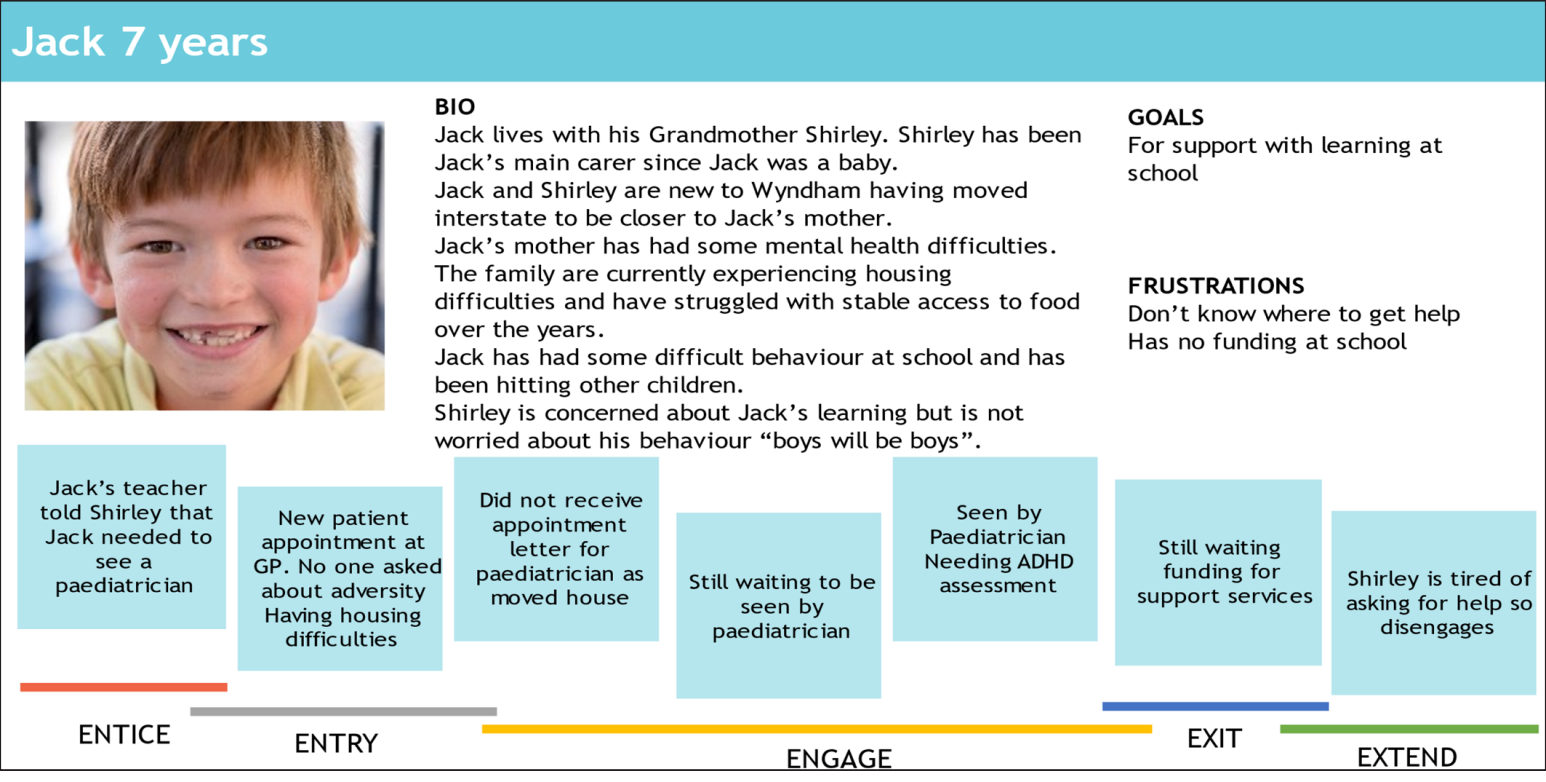
Persona and client journey map.

In human-centered design, a persona is a fictional character containing a composite of different accounts and experiences, created to represent the typical client’s interactions with the service or product [37,38]. Personas help to identify assumptions about users and to keep decision making and service development client focused. SL created three archetypal personas of child clients and their families through a combination of clinical observations and thematic analysis of qualitative interviews with particular attention to types of clients that were utilising the service (see example in Figure 2). The thematic analysis focused on the barriers for children and their family in accessing help for adversity. Different types of clients had different barriers, and this was used to inform and develop different personas that represented these different types of clients. The persona narratives and key quotes focused on physical attributes, background, attitudes towards the service and personal traits [39]. We also charted the key barriers to service access across a commonly used product design framework entitled the 5 Es of the client journey: entice, enter, engage, exit and extend [40]. These client personas and journeys acted as a launching pad for co-designing solutions and components of the Hub.

### Stage 4: Co-design workshops and consultations

The Child and Family Hub was co-designed through a series of workshops and consultations with intersectoral practitioners and families. A co-design team undertook seven full-day co-design workshops over 10 weeks focused on the client journey through the Hub. The workforce consultations focused on the workforce capacities and infrastructure required for the Hub’s implementation. Two community conversation sessions in a local shopping centre and a community centre were also held. Table 1 displays the topics, format and processes of the workshops and consultations.

### Co-design workshops

#### Team members (participants)

The co-design team of seven comprised two community members and five practitioners who lived in Wyndham Vale and/or worked in local health, family services, community and early childhood services (see Table 2). The practitioners held service delivery and strategic positions within their organisations and were those who responded to a flyer emailed by the local service networks (approx. *n* =35 organisations). Five practitioners in total applied for, and were selected, to participate. The two community representatives were parents with children aged 0–8 years who responded to an advertisement on the local council website inviting ‘families living with life challenges’ to join the team as community representatives. We selected the community members from a sample of 10 potential community members based on their experience of services in Wyndham and availability to attend all workshops in person. All team members provided informed written consent prior to the first workshop.

**Table 2:** Demographics of participants across all project stages.


PARTICIPANT TYPE	n	MEDIAN AGE IN YEARS	GENDER: FEMALE n (%)	ENGLISH MAIN LANGUAGE SPOKEN AT HOME n (%)	ABORIGINAL AND TORRES STRAIT ISLANDER STATUS n (%)

**FAMILY**					

**Interviews**	17	35–44	17 (100)	15 (88.2)	2 (11.8)

**Online consensus study**	2	25–34	2 (100)	0 (0)	0

**Co-design workshops**	2	25–34	2 (100)	1 (50)	0

**User testing in community conversations**	100	*	*	*	*

**TOTAL**	121	*	*	*	*

**PRACTITIONER**					

**Interviews**	26	35–44	25 (96)	26 (100)	0

**Online consensus study**	17	35–44	17 (100)	15 (88)	1 (5)

**Observation**	5	35–44	5 (100)	5 (100)	0

**Co-design workshops**	5	35–44	5 (100)	4 (80)	0

**Workforce consultation**	27	*	23 (85.2)	*	*

**TOTAL**	80	*	75(93.8)	*	*


* Not recorded.

#### Structure

**Figure 3:**
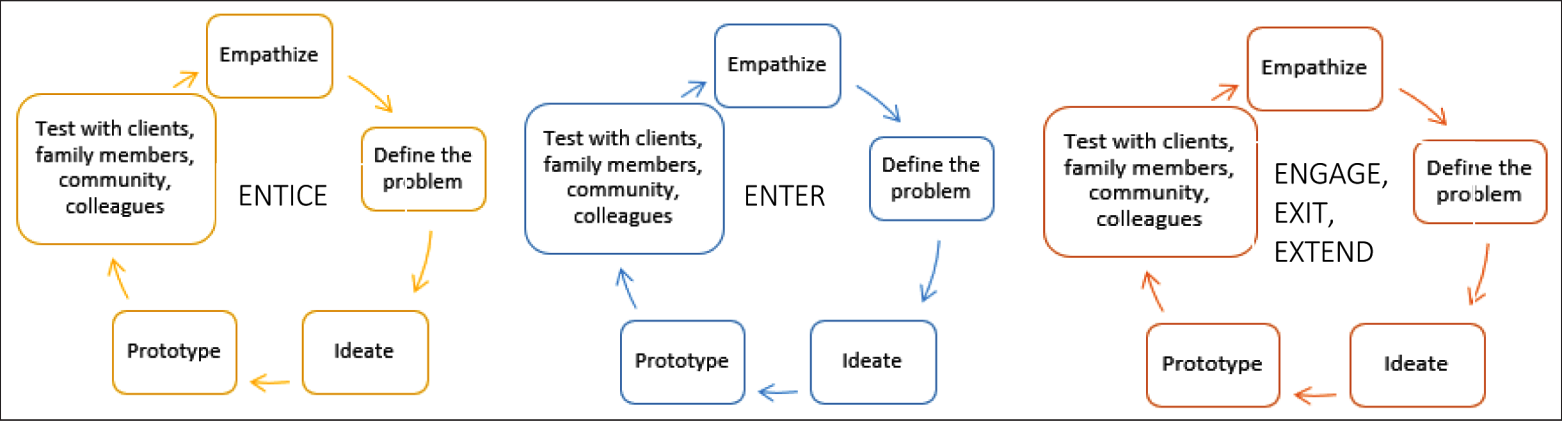
Co-design workshop structure.

The workshops were structured thematically around the 5 Es framework of the client journey: entice (workshops 1 and 2), enter (workshops 3 and 4), and engage, exit and extend (workshops 5, 6 and 7; see Table 1 and Figure 3). The co-design team had a one-week rest break between each E theme. During the workshops, each E theme was developed using a human-centred design process and tools: empathize, define the problem, ideate, prototype and test (see Figure 3) [40].

#### Preparatory scope setting and team building

Before the workshops, the two facilitators (TH, SP) provided the team members with a welcome booklet and had a one-on-one phone or virtual meeting to orient each team member to the project. The booklet specified what was in and outside of project scope, the workshop structure, the design process, and preparing to adopt a curious and flexible mindset. This content was then revisited in the first co-design workshop as a team. The team also agreed upon ways of working including being respectful and curious, and valuing diversity.

#### Empathise

Each E theme began by using the three personas and their respective client journey maps to understand the service touch points and empathise with typical family Hub clients. Using Post-it notes, the team worked together to map the key challenges across multiple levels of the service ecosystem (i.e., family, practitioner and system levels) at each E theme of the client journey. This mapping was important for visually representing the different stakeholder and system constraints impacting on services and in doing so generated empathy for both families and practitioners. We returned to these personas and journey maps for each E theme to ensure that generated solutions were needs-driven and feasible. In addition, guest speakers joined a session to provide detail on the current ‘entry’ process. The community representatives and practitioners also shared their/their clients’ experiences of navigating each step of the client journey.

#### Define the problem

The next step in the design process was to define the problem area through generation of problem statements (diverging process) followed by condensing and focusing on a shared understanding of the challenges to be addressed at each E theme (converging process) [29]. The ‘How Might We’ tool was used to transform the access challenges into problem definition statements. For example, the entice challenge of lack of awareness of available services became ‘How Might We raise awareness of the Hub in the community?’. The team independently generated numerous How Might We statements, displayed these on post-it notes, and team members independently voted on the most important/resonant statements to take forward into ideation. After voting, the facilitators worked with the team to cluster these problem statements thematically.

#### Ideate

The ideation step similarly involved diverging and converging independent and group processes using a round robin approach [29]. The team independently generated ideas for solutions to the problem statement themes, presented their ideas to the group which were strengthened through peer feedback and voted on these independently. The team then worked in smaller groups to create a detailed storyboard of the prioritised ideas on butchers paper or on Microsoft PowerPoint, i.e., a visual sequence of the steps and assumptions required to actualise the idea [40].

#### Prototype

The prototype step involved the team members working in small groups to create an early sample or model (e.g., videos, flyers, hardcopy materials, sketches) of each of the ideas developed for the 5Es of the client journey. The tangible physical or digital prototype is a design tool for increasing inclusivity by expressing insights that might not be captured in word descriptions alone [24].

#### Test

The final step of the design process involved testing the prototypes and storyboards with community members and practitioners. Between the workshops, team members were asked to present the prototypes to five or more of their family members, clients and/or colleagues who cared for children aged 0–8 years or worked with families with children this age. The co-design team members asked these stakeholders for their feedback on each prototype i.e., what they understood the prototype to be, what they liked, what could be improved. Microsoft Teams housed all outputs so that the team could collaborate between sessions and type the feedback they received into a standard feedback template. We also held two community conversations to engage the broader community. We created a pop-up stall at the local shopping centre over two days and a one-day stall at the community centre to meet families in familiar places. At each community conversation, team members tested the prototypes with caregivers while their children did a colouring-in activity and described how they would design the Hub’s ‘welcoming physical environment’. The drawings were retained by the research team with permission from the child and parents for analysis of the general components across drawings e.g., colourful setting, toys and activities, smiling staff, etc. Team members then relayed the user feedback on each prototype and reached consensus through group discussion on how to consolidate the prototypes.

### Workforce consultation process

In parallel, we conducted consultations with 27 health, social and family service practitioners of the service to elaborate and tailor the three fixed workforce elements of the Hub: workforce training and development, learning collaboratives and the community directory (see Table 1). During each consultation, SL presented the three fixed workforce elements and asked for feedback into the delivery and content of each element. The consultations were conducted with each practitioner group separately, mainly during team meetings when most staff were present. Practitioners had a strong preference for the training to occur over one day due to service constraints, so we adjusted the training accordingly. Consultations were also used to develop the new practitioner roles in the Hub and ensure that these roles met the expressed needs. When there was a range of preferences (i.e., the format and content of the community directory), we developed several user options.

### Reflections and outputs from the co-design workshops and consultations

HL and SL typed anonymised, detailed notes of each co-design workshop and consultation which included reflections by the team and the facilitators. The co-design workshops were evaluated using the 21-item Public and Patient Engagement Evaluation Tool (PPEET) [41]. The PPEET asked team members to rate the extent to which the workshops promoted a participatory culture and fostered collaboration. We commissioned a design agency to produce two journey maps illustrating how the personas moved through each prototype in the Hub. The team members also provided a short video or text response about their experience of the co-design process, which we compiled to place the team’s voices at the centre of future presentations. All digital data (i.e., audio recordings, detailed notes, digital prototypes) and hardcopy data (i.e., hardcopy prototypes, hand-written storyboards, etc) are stored securely at Murdoch Children’s Research Institute and will be destroyed at the end of the study period.

## Results

We present our key reflections on the process, principles, tools and challenges from the four project stages to co-design the Hub.

### Participants across all stages

In total, we engaged 121 family participants and 80 practitioners (see Table 2). Our sample was disproportionately female, spoke English as a main language and were aged 25 to 44 years old. Fewer family participants of interviews, the online consensus study and the co-design team (*n*=16, 76.2%) spoke English as their main language than practitioner participants of the same activities (*n*= 50, 95%).

### Processes

#### Trust building

Processes to engender trust were important across all stages. We conscientiously worked to build trust with community and service stakeholders during the governance and partnerships stage by transparently discussing the project scope (i.e., fixed and flexible inputs). Families’ mistrust of services emerged as a barrier to service engagement in the formative research. This meant that the co-design workshops focused on developing prototypes to build trust between the Hub and the broader community. Trust building was also crucial between team members in the co-design workshops to promote open expression in the creative process.

#### Team forming and building

Strategic team forming and building processes were necessary for creating a cohesive co-design experience. We reimbursed community representatives of the co-design team and advisory group to enable their participation and recognise their contribution. All practitioners but one contributed their time in-kind to the co-design workshop and consultations, and one organization took up our offer to backpay for a practitioner team member. We recruited co-design team members with a wide range of experiences, knowledge of Wyndham, and an openness to learn. As facilitators, we strove to foster a welcoming, inclusive space by acknowledging that the design process was a new way of working and uncomfortable at times. Team reflections on the co-design workshops explained how the design process helped to build a safe team dynamic:

*“When everyone is united around the cause and a safe platform is provided, the team forms just dynamically”* (Community #2, video)

*“[The strengths of the workshops were] clear goals and objectives for each workshop, openness to different ideas and perspectives”* (Practitioner #2, evaluation survey)

### Principles

#### Inclusion and diversity

All co-design team members rated all 21-items on the PPEET as ‘high’ or ‘very high’ agreement, indicating a strong satisfaction with the participatory culture of the workshops. This satisfaction was reflected in comments by the team members about the power of bringing community and practitioners together to generate mutual learning and provide diverse perspectives. Practitioner members described how the co-design workshops had improved their understanding of families:

*“One of the best things… was being able to work with different organisations in the community and also the community members. It was really valuable to hear the lived experiences from the community members and be able to work together in a really collaborative and friendly setting”* (Practitioner #2, video)

Several practitioners said the co-design process changed their practice to work more closely with families: “*personally, this process has now influenced the way I work in the future*” (Practitioner #1, evaluation survey). Community representatives also explained that it was helpful for them to understand the system constraints impacting on practitioners.

Working in a mixed stakeholder team added complexity. One community representative said it was difficult to express critical views of services despite facilitation encouraging this. Likewise, despite the improvement focus of the design process, some practitioners were threatened by the critique brought about through the ‘emphasise’ phase. In addition, several practitioners disclosed their lived experiences during the process. Hence, we followed up with all members after sessions.

#### Meet people where they are

Upon the recommendation of the co-design team, we undertook user testing in the local shopping centre and community centre instead of at the Hub. This was to meet families in places familiar to them, particularly given their barriers to health service access. The co-design team members also designed the colouring-in activity for children to “*capture the children’s voice”* (Practitioner #5, workshop notes). Our advisory group also recommended using existing local government networks to connect with practitioners and families.

### Tools

We used a range of design tools in our co-design process i.e., personas, How Might We statements, round robin ideation, storyboards, prototypes and user testing. Personas were an effective tool for building empathy for the Hub clients and integrating evidence into the design process. After some initial hesitation with using these tools for the first time, the co-design team easily transitioned from How Might We statements to prototypes in one day in the later workshops. Several team members said they liked the “hands-on” aspect of the design tools. One community member described how the structure afforded by these tools enabled her participation: “*It was very structured and non-confronting environment which made sharing my views easier.”* (Community #2, evaluation survey)

### Challenges

Resourcing (i.e., time, human resources) was a challenge to the co-design process. Stakeholder engagement was negatively impacted by the COVID-19 pandemic because Wyndham was a ‘hot spot’ for COVID-19 transmission and experienced increased family employment stress (102% increase compared to pre-COVID-19) [42]. This meant that families and practitioners were stretched, fatigued and had other priorities. We also had to exclusively engage stakeholders online which limited our engagement with community members in the early part of the project because we could not rely on ‘hanging out’ at community services or warm face-to-face connections with clients through Hub practitioners.

A substantial time investment was required to plan and execute the workshops and consultations. The facilitators met for half a day before and after each workshop for the 10-week period to plan and iterate the co-design workshop materials and process. All co-design team members said it was difficult to juggle their commitment to the project with other priorities (i.e., work and caregiving).

*“It was very intensive and I felt like I was neglecting my work. The turnaround times were a bit short to do the prototyping.”* (Practitioner #3, workshop notes)

*“I felt it was too long and it was a big commitment to lock yourself in to every session. Would have like to be able to drop in and drop out although I also see the value of being present for the whole thing”* (Community #1, workshop notes)

## Discussion

This paper fills an important methodological gap in co-design research with families experiencing adversity by describing the process, principles and tools used to co-design an integrated health and social care Hub.

### Processes and tools

Human-centred design offered a systematic process and tools for integrating formative evidence with lived and professional experience in the Hub’s co-design. We clearly specified how multiple forms of knowledge were merged in our co-design – both in this paper and to stakeholders during the process – to establish a shared understanding of the parameters and constraints of family and practitioner engagement. We did this by framing the co-design as comprising flexible and fixed inputs, and by defining ‘adversity’ and ‘co-design’ in the context of this project. This transparency around knowledge integration created the enabling environment for the project to accommodate innovation while also mitigating ‘scope creep’ in which attempts to address important but tangential community issues would have made the project unfeasible [24,43]. These findings have important bearings for future research and practice that aims to actualise Australian and international commitments to centre lived experience in mental health system reform [18,19]. Our study also demonstrates how co-design methods of engagement can bridge the research-practice gap through direct translation of evidence into service development [44].

The relational focus of the human-centred design process meant that community members and practitioners engaged on equal footing as team members [45]. As in previous co-design projects, we needed to iteratively negotiate the power differentials between our mixed stakeholder co-design team [13,46]. We reduced information asymmetry between practitioner and community members by focusing on the practitioner and system level barriers (i.e., the practitioner experience) alongside the client journeys [11]. Client personas were particularly effective as a storytelling device for generating empathy for Hub clients based on the formative qualitative research [13]. Personas were also key in adopting a family-centred approach to the Hub’s co-design i.e., our use of child personas placed children at the centre of the design process to acknowledge that we were working directly with caregivers. We also directly engaged children during the testing phase. Hence, our study adds methodologically to the literature of integrated care for families with young children which is crucial for achieving family-centred care [26].

### Principles

The co-design principles in this study align with eight guiding principles for effective community engagement that De Weger identified in a recent rapid realist review [47]. Table 3 maps our methods and strategies against these principles. In summary, we provided multiple avenues of participation to engage a diverse range of practitioners and community members that included children. This flexibility overcame differences in people’s preferences and abilities to participate. Reaching families in familiar places and through known networks acknowledged the importance of trust building processes with marginalized communities [22,48]. Hosting community conversations at the local shopping centre and community centre was also effective for garnering a large amount of user feedback in a relatively short period of time. Engaging these local stakeholders promoted ownership of the project and meant we avoided reliance on “super users” i.e., service users who become socialised to research processes through their repeated participation but whose perspectives may not reflect those of the most vulnerable families [24]. Hence, our project suggests that co-design research with families experiencing adversity in an integrated care context is best approached as a suite of resources rather than a “single off-the-shelf framework” [14].

**Table 3:** Rapid realist review identified eight guiding principles for effective community engagement in an integrated care setting [47].


PRINCIPLES FOR EFFECTIVE COMMUNITY ENGAGEMENT	OPERATIONALISATION IN CO-DESIGN OF CHILD AND FAMILY HUB

**(1) Ensure staff provide supportive and facilitative leadership to citizens based on transparency**	Transparent articulation of project scope (i.e., fixed and flexible inputs) and core project concepts (i.e., co-design and adversity) Transparent processes for progressing ideations to storyboards to prototypes using group decision making and independent voting

**(2) Foster a safe and trusting environment enabling citizens to provide input**	Use of existing community platforms for community members to find out about the project Recruitment of community representatives and participants in advisory group, formative research and co-design workshops through trusted community platforms

**(3) Ensure citizens’ early involvement**	Community members engaged from outset of the project during stakeholder engagement phase and later formative research phase preceding the active co-design workshops and consultations

**(4) Share decision-making and governance control with citizens**	Community members and community organisations represented on advisory group and co-design team Peer researcher member of research team

**(5) Acknowledge and address citizens’ experiences of power imbalances between citizens and professionals**	Iterative negotiation of power differentials between our mixed stakeholder co-design team Design process and tools engaged stakeholders on equal footing as team members Hands on, practical aspect of design tools enabled participation

**(6) Invest in citizens who feel they lack the skills and confidence to engage**	Preparatory scope setting and team building to support community members to meaningfully participate in the co-design workshops. Iterative checking in with community representatives on advisory group and co-design team to promote confidence and problem solve barriers to participation

**(7) Create quick and tangible wins**	Tangible prototypes rapidly developed and tested Rapid feedback on desirable and viable Hub prototypes

**(8) Take into account both citizens’ and organisations’ motivations**	Multiple avenues for participation to account for different levels of motivation and ability to participate in the project Personas captured child and family motivations for service usage Mapping of service and system barriers included practitioner motivations and experience of care provision


### Organisational context and resources

The community health partner’s (IPC Health) organisational capacity and focus on innovation was a key driver for the project. This created an authorising environment for service managers to carve out space for their staff members to participate and invest in facilitation. Such authorising environments have the power to catalyse co-design efforts in healthcare settings [13,49], or hinder progress when they are absent [24,45]. Even with supportive managers, however, practitioners on the design team still felt they needed to complete their routine workload, which is a barrier reported across studies [11,45]. Balancing the time commitment of co-design team members with the need to undertake additional user testing is a challenge in co-design projects [43]. Future research should investigate how different stakeholders can design different points of the client journey (to minimise the burden on co-design team members) whilst ensuring continuity of the design concept.

A significant investment was required to implement the co-design process. We allocated considerable time and project resources to ensure that the requisite project infrastructure (i.e., governance) and stakeholder engagement (i.e., local ownership of the project) was in place before we embarked on the active co-design workshop and consultation [45]. Resourcing our facilitators to manage, develop and implement the co-design process was also an important enabler, as has been shown in other studies [11,13,45]. Facilitation was important to avoid the loudest voices dominating the process and ensuring that there was respectful and psychologically safe conduct for members with lived experience (including both community members and practitioners) [17]. This resourcing decision recognised the “*emotional labour of working collaboratively*” [8].

### Strengths and limitations

The co-designed Hub may not adequately meet the needs of male caregivers and diverse communities because we primarily engaged with females who spoke English. User testing with male caregivers and through our team members who spoke languages other than English went some way to rectifying this gap. A second limitation is that we did not record detailed demographics of user testing; an oversight partly because we did not expect this tool to be so effective. Future research should explore platforms to effectively engage with male and diverse caregivers in user testing.

A key strength of this study was the detailed specification of the processes, principles and tools we employed, which allows for replication and learnings across contexts in accordance with principles of good community development and place-based approaches to integrated care [47]. Our study is also strengthened by the extensive engagement process of a range of stakeholders undertaken prior to commencing the co-design process. We also incorporated a validated participant evaluation using a standardised survey [50].

## Conclusion

The Child and Family Hub model was co-designed in partnership with families, intersectoral practitioners and community members. Human-centred design offered a systematic process and tools for integrating formative evidence with lived and professional experience in the Hub’s co-design. Applying community engagement principles meant that a diverse range of stakeholders were engaged across all stages of the project which built trust in and local ownership of the Hub model. Future co-design projects with families experiencing adversity in an integrated care context should develop strategies for language, stakeholder engagement, team composition and resourcing.
